# T145. ALTERATIONS IN SUPERFICIAL WHITE MATTER IN THE FRONTAL CORTEX IN SCHIZOPHRENIA: A DWI STUDY USING A NOVEL ATLAS

**DOI:** 10.1093/schbul/sby016.421

**Published:** 2018-04-01

**Authors:** Ellen Ji, Sarrazin Samuel, Marion Leboyer, Miguel Guevara, Pamela Guevara, Cyril Poupon, Antoine Grigis, Josselin Houenou

**Affiliations:** 1 INSERM; 2 University of Concepción; 3 Neurospin

## Abstract

**Background:**

Alterations in brain connectivity are strongly implicated in the pathophysiology of schizophrenia (SZ). Very recently, evidence is mounting to suggest that changes in superficial white matter (SWM) U-shaped short range fibers are integral components of disease neuropathology, a theory that is supported by findings from postmortem studies and less often in vivo in patients with SZ. This diffusion weighted imaging (DWI) study aimed to investigate SWM microstructure in the frontal cortex in people with SZ.

**Methods:**

Whole brain tractography was performed in 31 people with SZ and 54 healthy controls using BrainVISA and Connectomist 2.0 software. Segmentation and labelling of superficial white matter tracts were performed using a novel atlas characterizing 100 bundles. Principal Components Analysis (PCA) using a varimax orthogonal rotation was performed on mean generalised fractional anisotropy (gFA) of bundles located in the frontal cortex. Composites scores were computed for each subject, reflecting a linear combination of mean gFA values.

**Results:**

PCA revealed three components explaining 19.7 %, 5.8 %, and 5.4 % of the total variance. The mean score of the second component was significantly lower in the people with SZ compared with controls (p = 0.01) and included 13 bundles connecting regions in the pars orbitalis, insula, pars triangularis, pars opercularis, orbitofrontal cortex, anterior cingulate, superior frontal cortex and middle frontal cortex.

**Discussion:**

Our results support findings of reduced white matter integrity in the frontal cortex in people with SZ. Moreover, PCA may be helpful in identifying specific networks as the deficits do not appear to be widespread. Identifying patterns of superficial white matter dysconnectivity may be helpful in understanding the prominent symptoms and cognitive deficits and observed in SZ.

